# Antibiotic therapy impact on intravesical BCG therapy efficacy for high-risk localized bladder cancer treatment

**DOI:** 10.3389/fonc.2023.1240378

**Published:** 2024-03-08

**Authors:** Cécile Aubert, Thibaut Culty, Merzouka Zidane, Pierre Bigot, Souhil Lebdai

**Affiliations:** ^1^Urology Department, University Hospital of Angers, Angers, France; ^2^Pathology Department, University Hospital of Angers, Angers, France

**Keywords:** bladder, cancer, Bacillus Calmettes et Guerin, antibiotics, fluoroquinolones

## Abstract

Intravesical Bacillus Calmettes-Guerin (BCG) instillations is the gold standard adjuvant treatment for high and very high-risk non-muscle-invasive bladder cancer (NMIBC). Antibiotics may be required to treat asymptomatic bacteriuria before instillations or to prevent side effects. By modifying the bladder microbiota and through its bactericidal action, it could modify the efficacy of BCG. This study evaluates the impact of antibiotics received during BCG-induction treatment on the oncological outcomes for high and very high risk NMIBC. We retrospectively included all patients who received a full induction regimen of BCG therapy between January 2017 and June 2022. Clinical and tumor characteristics as well as tolerability were collected. Recurrence-free survival (RFS) and progression-free survival (PFS) were compared according to the prescription of antibiotics, its type and duration. A total of 126 patients were included, 86.5% of the tumors were high risk and 13.5% very high risk. The median follow-up was 31 months (7-60). 36% of the patients received antibiotics during BCG-induction treatment (among which 44% received fluoroquinolones). 21.4% of patients had tumor recurrence. There was no difference in RFS (p=0.902) or PFS (p=0.88) according to the duration or the type of antibiotics received. The use of a prolonged antibiotic treatment (> 7 days) significantly increased the duration of the BCG-induction treatment from 35 to 41,5 days (p=0,049) and the median number of delayed treatments by 1,5 [0-4]. Neither the use of antibiotics nor their duration modified the risk of recurrence or the intensity of side effects in multivariate analysis. Antibiotics received during BCG-induction immunotherapy did not influence oncological short-term outcomes or intensity of side effects.

## 1 Introduction

BCG therapy works by introducing the tuberculosis pathogen into the urothelial microbiota and stimulating the host’s innate and adaptive immunity against urothelial tumor cells. This immune stimulation induces the recruitment and the multiplication of immune cells effectors in the tumor microenvironment (1). The response rate to BCG therapy is variable and multifactorial (2). Performing systematic urine cytobacteriological examination (UCE) before each instillation is likely to induce antibiotic prescriptions which might induce bacteriological resistances and delay BCG treatments in case of positive UCE.

Recent studies suggest a potential impact of antibiotics in the host response to immunotherapy at a systemic and a urothelial levels (3–5). Pak et al, reported a significant decrease in recurrence-free survival (RFS) and progression-free survival (PFS) among patients who received 7 or more days of antibiotics, either concurrent or within 30 days prior to BCG induction, for high and very high risk NMIBC (6). They evaluated retrospectively 276 patients with T1 or high-grade tumors. 96 patients (34.8%) received a long course antibiotherapy (≥ 7 days) within 30 days prior to BCG therapy. Antibiotherapy for more than 7 days was independently associated with a higher risk of recurrence (HR: 2.45; 95% CI [1.49-4.05], p<0.001) and progression (HR: 3.68; 95% CI [1.65-8.22], p=0.001).

As a result, prolonged or repeated antibiotherapy may have an impact on BCG immunotherapy efficacy by inactivation of the bacillus (7) or by altering the bladder microbiota, or by disrupting the instillation protocol schedule (8).

The aim of this study was to evaluate the impact of antibiotics on the efficacy of BCG therapy as an adjuvant treatment for high risk NMIBC.

## 2 Methods

### 2.1 Population

In this retrospective study, we included all patients who received BCG therapy in a French university hospital between January 2017 and June 2022.

The study was approved by the local ethics committee (Study N°2022-089).

Inclusion criteria were: a confirmed high risk NMIBC according to the EAU classification (9) and completion of a BCG therapy induction regimen (6 BCG instillations).

Exclusion criteria were: TURB performed at another center, incomplete induction regimen, lack of post-induction cystoscopy, intermediate risk tumor, previous chemotherapy bladder instillation, or concomitant systemic immunotherapy.

### 2.2 Treatment and follow-up

All patients included underwent a complete tumor resection by TURB, followed by a full induction regimen of 6 weekly instillations. TURBT second-look was performed in cases of high-grade tumor (pTa or pT1) on the first resection, or in the absence of visualized muscle, according to current recommendations. Cystoscopy was performed 6 weeks after the end of the BCG treatment, and every 3 months during the first year. Maintenance treatment was performed according to the EAU guidelines (9). Antibiotics were prescribed in accordance with the protocol drawn up by the Infectious Diseases Committee of the French Urology Association (10).

Clinical characteristics were collected, including age at diagnosis, gender, smoking status, occupational exposure to urothelial carcinogens.

Tumor characteristics collected were: number of tumors, tumor size, Sylvester score, pathological grade, presence of carcinoma *in situ* (CIS).

We recorded antibiotic prescription before each treatment, including duration, and the therapeutic class received. The total duration of antibiotic treatment during induction was taken into account.

The number of UCE and delayed instillations during the induction treatment and during the maintenance treatment during the first year were quantified.

General and local side effects were reported by patients after each instillation, according to a self-administered questionnaire. The classification of the French Urology Association was used (11).

### 2.3 Endpoints

The population was divided into 3 groups according to the duration of antibiotic therapy: no antibiotherapy received, short course (2-6 days) and long course (≥ 7 days) antibiotherapy.

Recurrence was defined as the presence of urothelial cancer in the TURB anatomopathological analysis.

Progression was defined as the presence of a more aggressive tumor in the pathological examination of recurrence (grade progression, CIS occurence, T-stage progression).

Risk factors for recurrence were analyzed using a univariate and a multivariate analysis, including Sylvester score, previous recurrence, smoking, professional exposure, local and general side effects, antibiotherapy duration, and fluoroquinolone use.

### 2.4 Statistical analysis

Statistical analysis was performed using SPSS software (Statistical Package for the Social Sciences, version 15.0, Chicago, IL, USA). Categorical variables were reported in numbers and percentages and compared using a Chi-square test or Fisher’s exact test. The odds ratio was used to measure the relative risk. Quantitative variables were expressed as medians and standard deviations, and were compared using a non-parametric Mann Whitney test.

Survival analyses were performed using a Log Rank test (Kaplan Meier). Multivariate analysis was performed using logistic regression (Cox model), including covariates with a significance of p<0.20 in univariate analysis. For all analyses, p<0.05 was considered statistically significant.

## 3 Results

**Figure 1:**
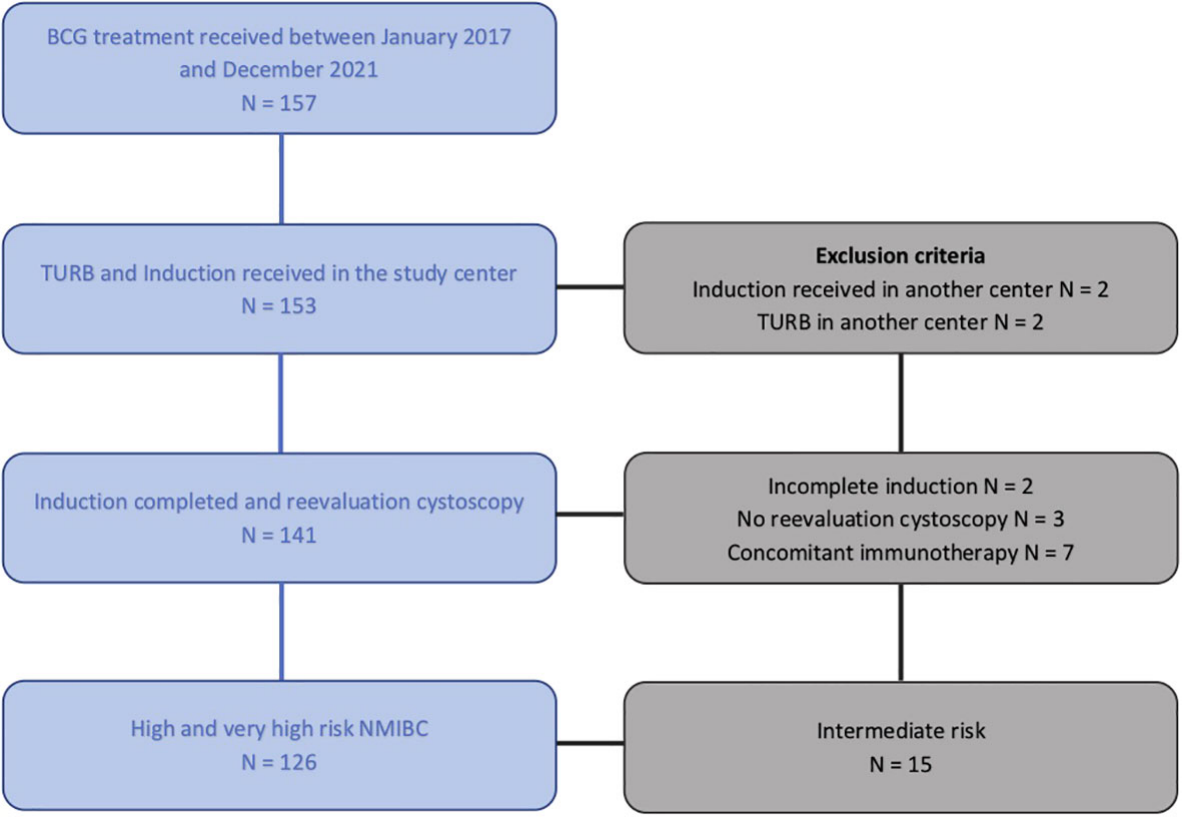
Flow chart. BCG, Bacille de Calmettes & Guérin; TURB, Trans Urethral Resection of the Bladder; NMIBC, Non-Muscle Invasive Resection of the Bladder.

Between January 2017 and June 2022, 157 patients received BCG therapy in our center. After applying exclusion criteria, 126 patients were included in the final analysis (**Figure 1**). Clinical and tumor characteristics are reported in **Table 1**. The median age was 71 years [35-90], 78% of the patients were males and 22% females.

**Table 1:** Baseline characteristics of patients and BCG treatment.

	Overall n = 126	No antibiotherapy	Short course antibiotherapy (2-6 days)	Long course antibiotherapy (≥ 7 days)	p
Total, n (%)		81 (64%)	25 (20%)	20 (16%)	
Age (median, yr.)	71 (35-90)	71 +/- 10,2 (35-90)	72 +/-10,7 (47-90)	70 +/- 10,9 (43-87)	0,583
Gender, n (%) Male Female	102 (81%) 24 (19%)	63 (77,8%) 18 (22,2%)	21 (84%) 4 (16%)	18 (90%) 2 (10%)	0,419
BMI (median, kg/m2)	26,4 (15,4-37,4)	26,7 (15,4-35,4)	26,1 +/- 3,6 (16,5-35,4)	22,6 +/- 4,5 (22,1-37,4)	0,525
Smoking, n (%)	93 (74,4%)	59 (72,8%)	21 (84%)	13 (68,4%)	0,434
Professional exposure, n (%)	7 (6,4%)	6 (7,4%)	0	1 (5,3%)	0,318
Pathologic T stage, n (%)					
Ta	50 (39,7%)	32 (39,5%)	8 (32%)	10 (50%)	
T1	66 (52,4%)	42 (51,9%)	16 (64%)	8 (40%)	
CIS	10 (7,9%)	7 (8,6%)	1 (4%)	2 (10%)	0,594
Tumor grade n (%)					
High risk	109 (86,5%)	68 (84%)	21 (84%)	20 (100%)	0,157
Very high risk	17 (13,5%)	13 (16%)	4 (16%)	0	
Tumor size, n (%)					
<3 cm	67 (58,3%)	42 (56%)	16 (69,6%)	9 (52,9%)	0,498
≥3 cm	48 (41,7%)	33 (44%)	7 (30,4%)	8 (47,1%)	
Number of tumors, n (%)					
Single	68 (54,8%)	49 (61,3%)	10 (41,7%)	9 (45%)	0,15
Multiple	56 (45,2%)	31 (38,8%)	14 (58,3%)	11 (55%)	
Concurrent carcinome in situ, n (%)	39 (31%)	24 (29,6%)	9 (36%)	6 (30%)	0,83
Prior reccurence, n (%)	25 (19,8%)	20 (24,7%)	3 (12%)	2 (10%)	0,184
BCG induction duration (median, days)	35 +/- 8,87 (28-99)	35 +/- 4,8 (34-56)	35 +/- 4,58 (34-50)	41,5 +/- 18,3 (28-99)	0,049
Systemic side effects					
None	70 (56%)	70 (56%)	9 (36%)	13 (65%)	
IA	27 (21,6%)	27 (21,6%)	5 (20%)	3 (15%)	0,084
IIA	20 (16%)	20 (16%)	9 (36%)	3 (15%)	
IIIA	8 (6,4%)	8 (6,4%)	2 (8%)	1 (5%)	
Local side effects					
None	20 (16,1%)	11 (13,8%)	4 (16%)	5 (26,3%)	
IB	71 (57,3%)	51 (63,8%)	12 (48%)	8 (42,1%)	0,34
IIB	18 (14,5%)	10 (12,5%)	6 (24%)	2 (10,5%)	
IIIB	15 (12,1%)	8 (10%)	3 (12%)	4 (21,1%)	
Staggered courses of treatment during induction (n, median)	0,46 +/- 0,8 (0-3)	0 +/- 0,7 (0-3)	0 +/- 0,65 (0-2)	1 +/- 1,1 (0-3)	0,009
Staggered courses of treatment during the first year (n, median)	0,68 +/- 0,98 (0-4)	0 +/- 0,8 (0-3)	1 +/- 0,81 (0-2)	1,5 +/- 1,45 (0-4)	0,015
Treatments instilled in the first year (n, median)	9,14 +/- 2,4 (6-12)	9,0 +/- 2,4 (6-12)	9 +/- 2,35 (6-12)	9 +/- 2,4 (6-12)	0,402
Protocol compliance at 1 year, n (%)	59 (48%)	40 (50%)	11 (45,8%)	8 (42,1%)	0,80

93 patients (74.4%) were smokers and 5% reported professional exposure to bladder carcinogens. In the overall population, 101 patients (80.2%) were included for a first tumor episode, 19.8% for a high-risk tumor recurrence. There was no difference in the rate of patients included for recurrence between the study groups.

The median follow-up was 31 months [7-60]. Pathological analysis revealed a pTa stage for 50 patients (39.7%) and pT1 for 66 patients (52.4%). According to the Sylvester classification respectively 86% and 14% of the tumors were at high and very high risk of recurrence, respectively. Finally, 31% of the anatomopathological analyses found CIS, alone or in association with another type of tumor.

14 patients (53.8%) recurred as high-grade tumors, 7% as pT2 tumors. 12 patients (46.2%) recurred as a low-grade tumor, possibly reflecting a partial response to BCG therapy. 37% of patients with recurrence had a low-grade tumor. The duration of antibiotic therapy received during induction BCG therapy did not correlate with the presence of high-risk tumor recurrence.

81 patients (64%) did not receive antibiotherapy during induction. 45 patients (36%) received at least two days of antibiotics during BCG induction. Of these, 20 patients (15.8%) were treated for more than 7 days. Tumor characteristics did not differ between the 3 groups. The median duration of BCG therapy induction was significantly longer when patients received antibiotics: 41.5 days [34-99] versus 35 days [34-56], p=0.049, respectively.

The median number of delayed courses during both induction and maintenance treatments were significantly higher for patients who received long course antibiotics compared to patients who did not receive any antibiotics: none vs 1 course during induction; p=0.009 and none vs 1,5 course after one year of treatment, p=0.015, respectively. These characteristics are summarized in **Table 1**.

**Figure 2:**
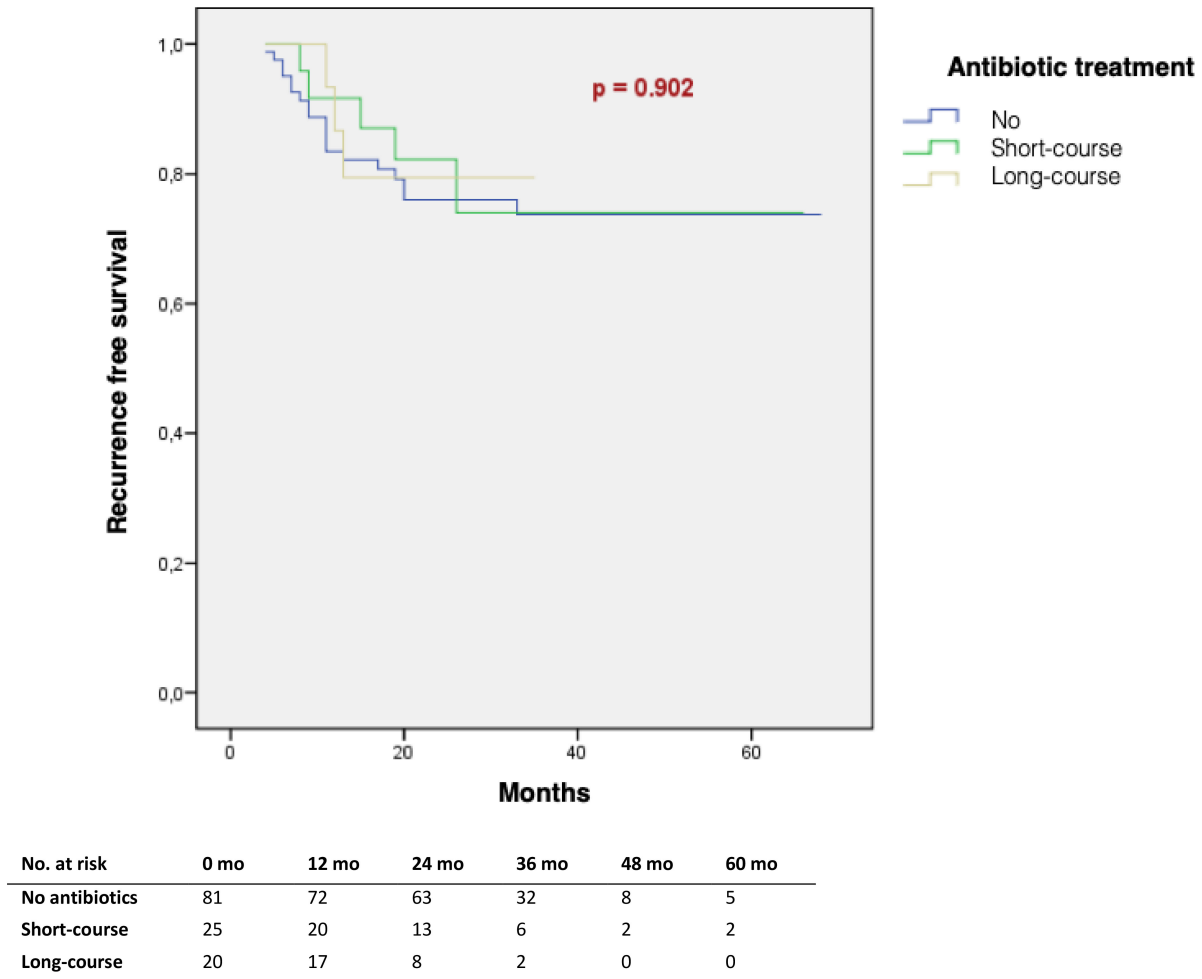
Tumour recurrence-free survival according to the total duration of antibiotic therapy received at induction (Kaplan Meier).

27 patients had a tumor recurrence, representing 21% of the total population. There was no significant difference in recurrence-free survival rates between patients who did not receive any antibiotics versus a short course antibiotherapy versus a long course antibiotherapy (p=0.902) (**Figure 2**). There was no significant difference in the progression-free survival according to the duration of the antibiotherapy (p=0.88). Only 10 patients (7.9%) showed tumor progression.

**Figure 3:**
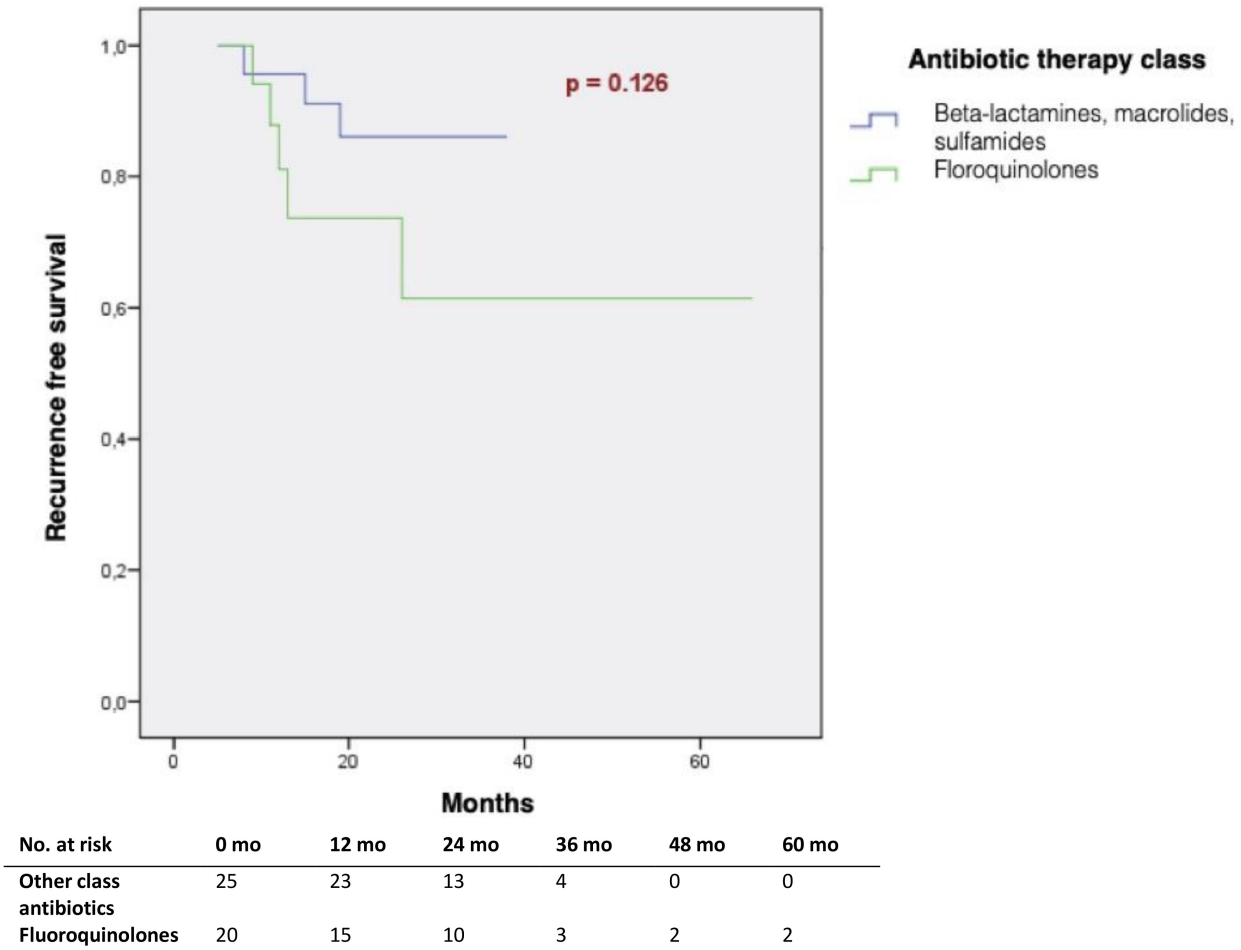
Tumour recurrence-free survival according to the class of antibiotic treatment received at induction (Kaplan Meier).

20 patients (44%) among those who received antibiotics during induction treatment, received fluoroquinolones. 5 (29%) of them relapsed at 3 years, compared to only 3 (12%) patients treated by another class of antibiotics. This difference was not significant (p=0.229) (**Table 2**). Among patients who received antibiotics, there was no difference in RFS, according to the class of antibiotics received (p=0.126) (**Figure 3**).

**Table 2:** BCG treatments caracteristics, according to the class of antibiotherapy received during induction. .

	Antibiotherapy during induction n = 45	Fluoroquinolones n = 20	Other class antibiotherapy n = 25	p
Systemic side effects				
None	22 (49%)	9 (45%)	13 (52)	
IA	8 (17,8%)	1 (5%)	7 (28%)	0,093
IIA	12 (26,7%)	8 (40%)	4 (16%)	
IIIA	3 (6,7%)	2 (10%)	1 (4%)	
Local side effects				
None	9 (20%)	6 (31,6%)	3 (12%)	
IB	20 (44,4%)	6 (31,6%)	14 (56%)	0,251
IIB	8 (17,8%)	3 (15,8%)	5 (20%)	
IIIB	7 15,6%)	4 (21,1%)	3 (12%)	
Staggered courses of treatment during induction (n, median)	0 +/- 0,9	1 +/- 1,05	0 +/- 0,64	0,006
Staggered courses of treatment during the first year (n, median)	1 +/- 1,14	2 +/- 1,25	0 +/- 0,75	0,002
Treatments instilled in the first year (n, median)	9 +/- 2,38	9 +/- 2,4	9 +/- 2,3	0,197
Protocol compliance at 1 year, n (%)		9 (20,9%)	10 (23,2%)	0,58

Very high Sylvester score and multifocal tumors were significantly associated with a higher risk of recurrence: OR=4.2 (CI[1.44-12.3]; p=0.01) and OR=3.1 (CI[1.26-7.62], p=0.010) respectively.

In multivariate analysis, a very high Sylvester score was independently predictive of recurrence, regardless of the duration and type of antibiotics received during induction. The intensity of local side effects appeared to be a protective factor (**Table 3**).

**Table 3:** Multivariable analysis for predicting risk of disease recurrence.

	Univariate	95% CI	P-value	Multivariate	95% CI	P-value
	Hazard ratio			Hazard ratio		
Smoking	3,63	0,942-12,98	0,061	2,24	0,772-6,54	0,137
Professional exposure	2,04	0,39-10,67	0,39			
Tumor grade (high vs. Very high)	4,2	1,44-12,3	0,01	3,12	1,34-7,24	0,009
Recurrent NMIBC	2,58	0,99-6,79	0,059	1,89	0,841-4,28	0,123
Antibiotic treatment						
None	Reference			Reference		
Short-course (2-6 days)	0,822	0,213-3,17	0,776	0,55	0,157-1,91	0,347
Long-course (≥7 days)	0,295	0,033-2,65	0,276	0,36	0,053-2,45	0,298
Fluoroquinolones antibiotherapy	4,72	0,91-24,54	0,065	2,10	0,73-6,057	0,167
Systemic side effects						
None	Reference					
IA	0,49	0,14-1,72	0,268			
IIA	1,61	0,295-8,78	0,582			
IIIA	NA	NA	NA			
Local side effects						
None	Reference					
IB	0,159	0,056- 0,451	0,007	0,265	0,116-0,605	0,002
IIB	0,048	0,005-0,468	0,023	0,072	0,009-0,568	0,012
IIIB	0,064	0,007-0,610	0,028	0,114	0,014-0,905	0,040

All factors with p<0.1 in univariate analyses were included in the Cox multivariate analysis. CI, confidence interval.

The duration of antibiotic therapy during induction was not correlated with the severity of local (p=0.34) or systemic (p=0.084) side effects. Fluoroquinolones treatment during induction did not significantly reduce the severity of local (p=0.251) or systemic (p=0.093) side effects compared to other antibiotic treatments. There was no association between the severity of side effects and compliance with the recommended protocol during the first year of treatment.

In univariate and multivariate analysis, the intensity of local side effects was associated with a lower risk of tumor recurrence. The hazard ratio was 0,072 (CI[0,009-0.568]; p=0.012) for side effects lasting from 2 to 7 days, and 0,11 (CI[0,014-0.905]; p=0.04) for those lasting more than 7 days (**Table 3**).

## 4 Discussion

In this study, the use of antibiotics during BCG induction treatment was not associated with tumor recurrence. Recurrence-free survival or progression-free survival in patients with high or very high risk NMIBC were not affected by antibiotherapy duration. This is a very important and reassuring finding considering that antibiotics (especially fluoroquinolones) are commonly used during BCG therapy either to treat asymptomatic bacteriuria before instillation or to prevent side effects.

In 2021, Pak et al. reported quite different results that have to be further discussed (6). Their main outcome was that antibiotherapy for more than 7 days concurrently or within 30 days before starting BCG therapy was found to be an independent risk factor for recurrence and tumor progression. The fact that they included patients who received antibiotics 30 days before the treatment has to be kept in mind especially because the proportion of patients who actually received long course antibiotics during the BCG induction treatment was very low. Furthermore, their study suffered from several biases. The authors did not distinguish the different classes of antibiotics which might be an issue considering the variability of the sensitivity of BCG strains depending on the antibiotics (7). Additionally, only 17.7% of the patients received a long course antibiotherapy concomitantly with BCG therapy: this should have been the main point of focus since the induction period is presumably the most critical period of BCG efficacy considering the high risk of interaction between the antibiotics and BCG and the risk of delayed instillations and protocol deviations. Furthermore, only 44.9% of the patients had received maintenance therapy: this might have been a major bias interfering with the risk of recurrence. Finally, the authors did not specify whether the antibiotic therapy received was during induction or maintenance period. All these biases contribute to a very heterogenous population with very different situations pooled together which make it very difficult to draw precise conclusions.

In our study, we investigated the antibiotic treatments received during induction treatment, in a homogeneous population with a complete BCG regimen and took into consideration the variability of BCG’s sensitivity to antibiotics by studying the different classes of antibiotherapy received.

In order to identify good BCG therapy responders, the susceptibility of BCG strains to different classes of modern antibiotics was studied (12). BCG strains were found to be very highly susceptible to fluoroquinolones. But *in vitro*, BCG appeared to be naturally resistant to pyrazinamide, cycloserine, beta-lactams, macrolides, nitrofurans and trimethoprim.

Regarding the use of fluoroquinolones, Damiano et al, reported in 2009 that the use of prulifloxacin to prevent BCG-related side effects did not affect the risk of recurrence at 6 months (13). A randomized controlled trial of the EORTC evaluating the concurrent use of isoniazid during BCG therapy found no impact on recurrence and tumor progression.

(14). These results are consistent with ours and contradict the hypothesis of a direct bactericidal effect of the antibiotics on BCG.

The bladder environment and the role of the urinary microbiome on bladder oncopathogenicity appears to be important to understand the inter-individual variability in response to BCG (15). The presence of different types of bacteria in the bladder microbiota could influence the immune response to BCG therapy. 16S rRNA sequencing of urine from patients with MIBC and NMIBC and from healthy volunteers has identified higher levels of bacteria such as Serratia, Escherichia-Shigella, and Pseudomonas in good responders to BCG (16, 17). These studies are limited by difficulties in collecting and identifying the urine microbiome with a high heterogeneity of bacteria detected. No study has yet identified a direct link between a decrease in the presence of these bacteria and a reduced efficacy of BCG therapy. In March 2022, the protocol of a prospective observational study investigating the role of the urine microbiome on the response to BCG therapy was published (18): these results will be a major step forward in better understanding BCG mechanisms.

According to a 2017 meta-analysis published by Poletajew, the presence of an asymptomatic bacteriuria did not increase the risk of urinary tract infection after BCG instillation. No correlation was found between the recurrence free survival and progression free survival (19). Antibiotic therapy received during the BCG induction did not appear to be associated with oncological outcomes. Our results support screening for and treating asymptomatic bacteriuria before BCG instillation. Nevertheless, the risk of multidrug-resistant bacteria selection remains high.

There is no evident correlation in the literature, between the importance of local side effects and the reduction of recurrence. Colombel et al, reported that routine use of prophylactic fluoroquinolones, did not increase the risk of recurrence or progression at one year (20). A recent randomized controlled trial reported a comparable compliance rate with the routine use of fluoroquinolones for side-effects prevention, and no significant reduction in local and systemic side effects for any grade (21). In our study, the median number of courses received at 1 year was 9.1, compared to the 12-course regimen recommended by the French urologic association during the first year (10). This deviation from the protocol may be related to cytobacterial urine examination, urinary functional signs or macroscopic hematuria. The recent shortage of BCG has also affected this pattern (22). Lamm et al. reported that 80% of patients discontinued BCG therapy due to increased side effects in comparison to the recommended protocol (23). In our study, only 48% of patients had a compliant regimen at one year. Patients who received a long course of antibiotics at induction had a significantly higher number of staggered courses during induction and at one year of treatment. This increase did not appear to have any influence on the survival outcomes. These results align with the lack of data to define the optimal BCG therapy, with current recommendations proposing an arbitrarily defined regimen (24).

Our study has several limitations. It is a retrospective, mono-centric study, with a limited number of events, decreasing the statistical power of the results. The lack of standardized protocols for antibiotics prescription may have resulted in a selection bias.

In our study, 44% of patients who received antibiotic therapy during induction received fluoroquinolones, without altering the intensity of general or local side effects. This contrast with the ITB01 study, which suggested that 200 mg fluoroquinolones administered 6 and 18 hours after instillation decrease local grade II adverse events (20). The recently published recommendations of the French urology infectiology committee, following the EAU recommendations for the management BCG therapy side-effects, no longer mention the use of fluoroquinolones for prophylaxis or curative purposes (8, 25).

As the action of intravesical BCG is based on the stimulation of anti-tumor immunity, treatments aiming at reducing its side effects must target this mechanism, in particular by using anti-inflammatory drugs or corticosteroid therapy.

## 5 Conclusion

The use of antibiotic therapy during induction of adjuvant BCG therapy did not influence recurrence-free survival or progression-free survival of a high-risk localized bladder cancer.

Long-course antibiotic therapy leads to an increase of the duration of BCG induction therapy, and a shift in the number of courses received in one year of treatment, without impacting the intensity of side effects.

## Data availability statement

The original contributions presented in the study are included in the article/supplementary material. Further inquiries can be directed to the corresponding author.

## Author contributions

CA and SL: project development, data collection, data interpretation, manuscript writing. TC, MZ, and PB: data interpretation, manuscript reviewing. All authors contributed to the article and approved the submitted version.
